# More Than a Diamine Oxidase Inhibitor: L-Aminoguanidine Modulates Polyamine-Related Abiotic Stress Responses of Plants

**DOI:** 10.3390/life13030747

**Published:** 2023-03-09

**Authors:** Zoltán Márton Köhler, Ágnes Szepesi

**Affiliations:** 1 Department of Biochemistry, Albert Szent-Gyorgyi Medical School, University of Szeged, H-6720 Szeged, Hungary; 2 Department of Plant Biology, Institute of Biology, Faculty of Science and Informatics, University of Szeged, Közép fasor 52, H-6726 Szeged, Hungary

**Keywords:** aminoguanidine, plant, nitric oxide synthase inhibitor, polyamines, copper amine oxidase

## Abstract

L-aminoguanidine (AG) is an inhibitor frequently used for investigating plant abiotic stress responses; however, its exact mode of action is not well understood. Many studies used this compound as a specific diamine oxidase inhibitor, whereas other studies used it for reducing nitric oxide (NO) production. Recent studies suggest its antiglycation effect; however, this remains elusive in plants. This review summarises our current knowledge about different targets of AG in plants. Our recommendation is to use AG as a modulator of polyamine-related mechanisms rather than a specific inhibitor. In the future overall investigation is needed to decipher the exact mechanisms of AG. More careful application of AG could give more insight into plant abiotic stress responses.

## 1. Introduction

Plant physiological responses during growth and development or under stress conditions are really complex, and many signal pathways work together to elicit a particular response. Studying the role of some important enzymes in these processes is crucial in order to develop our crop plants to be more resistant or tolerant to changing climate conditions. Mutant or transgenic plants could greatly enhance our knowledge about the importance of an enzyme in a specific physiological response. However, the fact that we only have the opportunity to use mutants in the case of a few plant species hinders the investigation of enzyme function. One of the strategies for investigating the role of an enzyme in plant abiotic stress processes is to use pharmacological inhibitors, compounds with different features. It is a huge task to decipher the exact mechanism of these inhibitors. Despite their many side effects, inhibitors are compounds frequently used for studying enzyme activity. L-aminoguanidine (AG) was described as a DAO or iNOS inhibitor, and its roles and potent mechanisms in animals and plants were characterized [1]. AG was first implicated in polyamine (PA) catabolism as it is involved in the diamine oxidase or copper amine oxidase (DAO or CuAO, EC 1.4.3.22) inhibitor mechanism, but experiments in animals also suggested that AG inhibits other processes, for instance, the inducible nitric oxide synthase (NOS) enzyme activity and the synthesis of advanced glycation end products [1]. AG is an amino derivate of guanidine, which may have a high affinity for amine oxidases, maybe due to stabilization by conformational changes or by forming a Schiff base with the catalytic center of the enzyme. Using AG as an inhibitor is of particular importance, but there are many side effects during its application, and sometimes, the results are highly controversial. The following two decades provided experimental evidence of the complex effects of this compound, with distinct pharmacological activities related to PA homeostasis and associated signaling pathways. However, the direct mechanisms behind the inhibitor effects of AG in plants are not fully understood. Thus, this study is aimed to review the plant-specific inhibitory effects of AG on PA homeostasis and related processes and to summarize the pathways that are related to PA metabolism in plants to characterize the widespread mechanisms of AG. We will discuss the effect of AG (i) as a DAO inhibitor affecting PA catabolism, (ii) as a hypothetical NO synthase inhibitor reducing the NO content in plant tissues, and (iii) as an antiglycation agent, which is not well-understood in plants. This compilation of our current knowledge could help form a more complete view of the efficiency of AG in plants, contributing to the more precise application in case of the duration, time point, and concentration of this compound in the future. 

## 2. DAO As the Main Inhibition Target of AG in Plants

PAs are essential N-containing polycationic compounds in all living organisms and important hub molecules which are responsible for connecting certain metabolic and hormonal pathways [2,3]. These molecules can exist in free, bound, or conjugated form. The three most important free PAs in plants are diamine putrescine (Put), triamine spermidine (Spd), and tetramine spermine (Spm). Some other important Pas, such as thermospermine (T-Spm) or cadaverine (Cad), are also known. Bouchereau et al. also suggested that PAs have important roles in plant development and plant abiotic stress-related processes [4]. Since then, multiple reports have shown significant roles of PAs in developmental processes, such as stomatal closure, xylem development, reproduction or programmed cell death, and abiotic stress responses, for instance, in salinity, drought, or heavy metal stress [5,6,7]. Recently, the role of PAs as signaling molecules involved in leaf senescence was described in plants [8]. Moreover, molecular mechanisms of PA-induced abiotic stress tolerance are considered important strategic targets for enhancing abiotic stress tolerance of agriculturally important crop plants [9], which underlines the importance of their proper understanding. 

**Figure 1:**
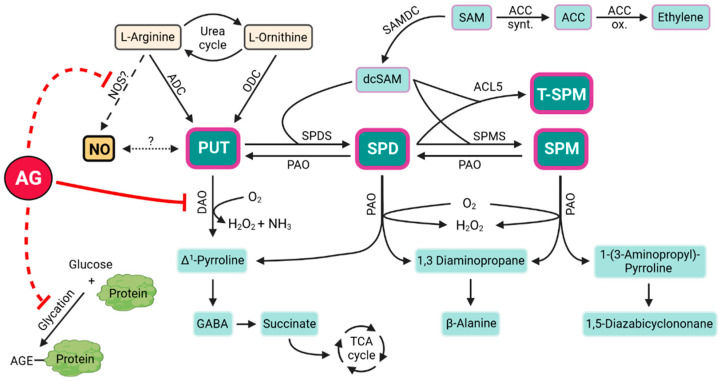
The potential AG targets of polyamine catabolism and its related mechanisms in plants. Dashed line shows that the connection between NO and Put is yet unknown, and the existence of the NOS enzyme is also questionable. The figure was created using BioRender.com. ACC: aminocycloparopane carboxylic acid, ACC synt.: ACC synthase, ACC ox.: ACC oxidase, ACL5: T-Spm synthase, ADC: L-arginine decarboxylase, AG: L-aminoguanidine, AGE: advanced glycation end product, DAO: diamine oxidase, dcSAM: decarboxylated SAM, GABA: gamma amino butyric acid, NOS: nitric oxide synthase, ODC: L-ornithine decarboxylase, PAO: polyamine oxidase, PUT: putrescine, SAM: S-adenosylmethionine, SAMDC: SAM decarboxylase, SPD: spermidine, SPDS: Spd synthase, SPM: spermine, SPMS: Spm synthase, TCA cycle: tricarboxylic acid cycle, T-SPM: thermospermine.

The main functions of PAs in plants, their metabolism, and homeostatic regulation warrant consideration of PAs as essential biogenic amines. Their properties were summarized and reviewed in a recent publication by Chen et al. [10]. PAs may exist in different forms, and these can be dynamically converted back and forth by enzymatic reactions, contributing to optimal PA homeostasis in cells [11]. The biosynthesis of Put could occur in two ways: from L-arginine (Arg) by arginine decarboxylase (ADC, E.C. 4.1.1.19) in many plants or from L-ornithine (Orn) by ornithine decarboxylase (ODC, EC 4.1.1.17) in some plants such as tomato. It is interesting to note that the main plant model organism, *Arabidopsis thaliana*, contains only the ADC pathway for the biosynthesis of Put. The synthesis of Spd is catalyzed by Spd synthase, whereas Spm is synthesized by Spm synthase. Besides the biosynthesis of these molecules, PA catabolism is another of the most important processes maintaining optimal PA homeostasis in plant cells. Oxidative PA catabolic reactions are mediated by two enzymes, the copper-containing DAO and the flavin-dependent polyamine oxidase (PAO, EC1. 5.3.11) [12,13]. DAOs can exist in dimeric form and can contain a copper ion and a redox-active cofactor 2,4,5-trihydroxyphenylalanine quinone (TPQ) in each monomer. DAOs can play a role in the intra- or extracellular degradation of amines, including mono- or diamines and PAs. These reactions cause terminal catabolism of these amines, mainly to diamine Put and Cad. Additionally, it is worth mentioning that there is another pathway, the so-called back conversion, which can support the conversion of higher PAs, such as Spd and Spm, back to diamine Put, ensuring the fine-tuned regulation of the optimal PA cycle. Localization of DAOs is primarily in apoplast and peroxisomes. DAOs show a broad substrate specificity, and their gene expression patterns have also been extensively investigated in the latest years. In *Arabidopsis thaliana*, ten different genes encoding DAOs were described, but only eight of them are functionally active. The ensuing enzyme reactions contribute to the production of hydrogen peroxide (H_2_O_2_) and some reactive aldehydes (Figure 1). 

PAOs oxidize higher PAs, such as Spd or Spm, resulting in amine derivatives, 1,3-diaminopropane, or 1-(3-aminopropyl)-pyrroline, as well as H_2_O_2_ [14]. The presence or absence of H_2_O_2_ can regulate other mechanisms in plant cells acting as signaling molecules or prooxidants. H_2_O_2_, as the major by-product of the PAO-catalyzed PA oxidation reaction, acts as the inducer of defense gene expression or cell wall stiffening and lignification [15]. It is important to consider the exact role of PA catabolism in abiotic stress tolerance because PA catabolism is involved in a diversity of plant functions [16], e.g., stomatal closure, responses to biotic stresses, or some abiotic stress situations, such as wounding or the effects of salt and heavy metal stress conditions. DAOs can act not only as PA-level modulators but as a source of bioactive compounds as well. Additionally, some authors suggest that DAOs could be potential checkpoints in the balance of PAs/H_2_O_2_ ratio to turn processes for survival or cell death. In a recent study, it was shown that a peroxisome-localized CuAOζ, which is highly expressed in cortical cells, and the reactive oxygen species (ROS) derived from CuAOζ are essential for lateral root development in *A. thaliana* [17]. Another publication revealed that PA catabolism, especially DAO activity, is critical for shoot morphogenesis in Brassica juncea. 

The actual inhibitory effects of AG on PA degradation have crucial importance in plants. Padiglia et al. described the different inhibitors of plant DAOs; however, it is important to pay attention to the safety of the application of these inhibitory compounds [18]. Table 1 summarizes the experimental conditions in the most important references where AG was used as a DAO inhibitor in plants. It can be seen that the AG concentrations used, as well as the plant species studied, and the treatment conditions applied were highly diverse. Conclusively, in order to detect any changes in plant growth and development related to AG, small concentrations of AG were effective [19,20,21], whereas, in some stress types, DAO inhibitory effects were induced by larger AG concentrations [22,23,24]. It is important to note that only a few studies used the same concentrations of AG under the same conditions, and this makes it difficult to compare the effects of this inhibitor. 

DAOs strongly connect to one of the most important degradation products of PA catabolism, namely, gamma-aminobutyric acid (GABA), which is strongly evidenced to be a signaling compound in plants. Recent studies also highlight the role of GABA in pulses because of its presence at high concentrations [31,32]. Pulses have received increasing attention in recent years because they can be considered functional foods due to their high GABA content [33]. In fava beans, AG inhibited DAOs at high concentrations (5 mM) with a simultaneous reduction in GABA levels, indicating a strong relationship between PA catabolism and GABA production [24]. Interesting evidence was provided by Hidalgo-Castellanos et al. [30], when AG was used to inhibit the DAO enzyme in *Medicago truncatula* to investigate the role of PA catabolism in nodulation. An amount of 1 mM AG was sufficient to completely inhibit the nodulation process of this leguminous plant species. AG alone significantly reduced the contents of some amino acids, for example, GABA or Arg, but during salt stress, these parameters showed higher levels. This biotic interaction between *Medicago truncatula* and Sinorhizobium meliloti during salt stress supported evidence that H_2_O_2_ produced by PA catabolism was responsible for symbiosis formation by nodules and increased. Put levels could act as a mechanism to reduce ammonium stress in *Medicago truncatula*, providing evidence of the urea cycle and PA metabolism in plants [30]. Evidence of the actual mechanisms through which AG influences PA catabolism and GABA synthesis still remains to be proved. A holistic experimental approach is required to decipher the complexities of AG effects. Legocka et al. [26] used AG as a DAO inhibitor to quantify the contribution of Put catabolism to proline (Pro) and GABA production in seedlings. They found that AG could reduce GABA content by 22%, indicating that the PA degradation pathway was included in GABA formation. Foliar applications of degradation products of PAs, such as GABA, can reduce stress levels following exposure to triazine herbicides, suggesting future uses of these compounds in agriculture [34,35]. An interplay was proposed between peroxidases, amine oxidases, and lipoxygenases in wound-induced oxidative bursts in green pea (*Pisum sativum*) seedlings [36]. A recent study reviewed the regulatory role of phytohormones and PAs and demonstrated plant stress responses by altering the GABA pathway [15]. Despite their importance, the concrete actions of AG on the GABA shunt and the related coordination of signaling pathways of PA degradation remain elusive in plants. 

Some evidence shows that AG affects PA catabolism in transgenic plants. Tobacco (*Nicotiana tabacum*) plants transformed with the oat ADC cDNA showed high DAO activity, and its oxidation reaction products could contribute to the detrimental effects. Using AG in this experimental setup caused necrotic symptoms in control plants and, finally, caused their death [37]. However, in soybean (*Glycine max*) cotyledons, AG induced plant defense when it was applied together with sodium chloride [38]. Using 2 mM AG successfully induced cold tolerance by PA oxidation [39]. The importance of PA catabolism and related phytohormonal changes is well established in plants, but our knowledge about its background is limited. The role of DAO activity was described as a source of H_2_O_2_ in abscisic acid (ABA)-induced stomatal closure in fava bean (*Vicia faba*) and suggested the role of calcium in this process [40]. PAs act as hub molecules under drought, and other abiotic stress factors are interconnected by NO and ABA [41]. The signaling pathways of PAs can upregulate NO production and promote ABA synthesis, which plays a crucial role in abiotic stress tolerance in plants [42]. CuAO mutant *A. thaliana* plants, *cuao1-1* and *cuao1-2*, are impaired in NO production, suggesting a function of CuAO1 in PA, ABA, and ABA-related NO production in plants [43]. Many studies focus on these molecules and their catabolic pathways, as they are strongly associated with the ROS metabolism [44]. In the next section, we summarize our knowledge about the effects of AG on NO biosynthesis in plants.

## 3. Disruption of NO Biosynthesis by AG in Plants

The second most studied effect of AG is the reduction of NO levels by inhibiting the NO synthase enzyme; however, the exact mechanism of this effect is not well understood and defined. Early studies proposed that the role of AG is to inhibit enzymes that possess carbonyl groups as a cofactor, e.g., NOS or semicarbazide-sensitive DAOs because it has a structure similar to the guanidium group of Arg [45]. Despite the enormous number of excellent articles that can be found in the literature about the NOS-like nature of plant NOS, no experimental evidence of the structure of plant NOS is available up to now [46,47]. NO is a gaseous molecule with a complex range of roles in plant development and stress response signaling pathways [48]. NO can act as a signal molecule in key developmental processes, such as senescence, flowering, germination, or photosynthesis. Additionally, this molecule is involved in processes associated with different environmental stress conditions (salinity, drought, flooding, heavy metal, temperature stress) or biotic stress factors. NO can be generated by a reductive and an oxidative route. The reductive route is more common in plants and more defined than the other. This route is involved in NO synthesis from nitrite by nitrate or nitrite reductase and involves the mitochondrial electron transport chain. The oxidative pathway of NO biosynthesis involves nitric oxide synthases; however, their exact structure is not well known. PA metabolism is strongly connected to the oxidative route of NO synthesis. The biosynthesis of both NO and PAs has a common source, as both can be generated from Arg. This raises the question of their competitiveness in some physiological responses. NO can induce PA synthesis by regulating PA biosynthetic enzymes such as ODC [49]. Studied in *Arabidopsis thaliana*, PA biosynthesis and NO are strongly interlinked [42], as NO formation increases in response to exogenous PA, and in turn, PA metabolism can be adjusted in a dose-dependent manner by exogenous NO [6,42]. Moreover, Arg is a precursor of both PAs and NO, and arginase has been considered a decisive checkpoint to direct the metabolism of Arg to either PAs or NO. Some amino acids (e.g., methionine), which are precursors of PAs, can affect the cellular status of NO [50]. 

In a recent study, a *cuao8* mutant was identified in *Arabidopsis thaliana*, and the resulting defect in NO biosynthesis related to inhibited DAO activity was proved by AG [20]. These observations indicated that enhanced arginase enzyme activity contributes to impairments of NO production and affects Arg pools in *cuao8* mutant plants [20]. The arginase pathway can impact the Arg level and, therefore, the substrate availability of NO production, in addition to oxidative and reductive NO biosynthetic pathways.

Several lines of evidence demonstrate that AG inhibits inducible NO synthase (iNOS) in mammals [51]. Although candidate NO synthases have been suggested in plants and NOS-like activities have been identified, no NOS homologs are present in plant genomes [46,47]. Moreover, numerous reports associate NO with plant development and stress responses, but no agreement has been reached regarding the origin and level of NO in different tissues. Most likely, NO metabolism is regulated and modified via multiple interconnected and overlapping pathways in plants [52,53]. 

The use of various concentrations, duration of treatment, and plant tissue models will likely produce evidence of different inhibitory effects of AG (Table 2). 

The concentration used is one of the most crucial factors in the pharmacological approaches of AG research. A concentration-dependent range of effects affecting other different signal pathways can be suggested. For example, in the crude extracts of *Nicotiana tabacum*, AG was used at a small concentration (0–50 µM) [54], whereas in the hypocotyl of sunflower seedlings (*Helianthus annuus*), 5 mM AG was applied to inhibit NOS activities [56]. Studying temperature of stress-induced effects in pepper leaves, the authors used a pharmacological approach to investigate the potential NO sources in these tissues [54]. They used 1 mM AG in experiments testing AG as an animal NO synthase inhibitor, and they found that Arg-dependent NO synthase activities were among the major contributors to NO production in pepper leaves. Despite the profuse evidence regarding AG-induced NO synthesis inhibition during abiotic stresses, it remains unclear how AG is able to inhibit NO biosynthesis during plant developmental processes. Based on the evidence, another question is how AG can induce NO reduction and whether that can occur by modifying the potential NOS enzymes or by indirectly affecting the level of NO donor molecules, such as Arg. Similarly, Astier et al. [46,47] emphasized the importance of adequately dosed applications of NO synthesis inhibitors in plants since the success of certain pharmacological approaches depends on the side effects of the inhibitor compound used, the concentrations applied, and the plant organs investigated. Therefore, the pleiotropic effects of AG and the dose concentrations also need to be carefully considered in future plant-related research experiments. It is also important to consider the simultaneous study of the different AG-related effects in order to demonstrate the importance and ratio of these responses. More studies need to be conducted to decipher the complex mode of action of AG in various plant species. 

It is suggested that there is a special interplay between PA metabolism and NO signal pathways in plants. This interplay could have a role in some environmental stress conditions, such as salinity or drought. Modern biotechnological and metabolomics approaches may produce insights into the PA–NO interplay. For example, chemical labeling could be used to identify and enrich reactive Arg residues in proteins [61]. However, the pathway through which AG influences NOS-like activities and NO–PA interactions in plants remains unknown.

## 4. AG and Its Possible Inhibitory Effects on Protein Glycation in Plants

The least known effect of AG in plants is the potential antiglycation process. Here we compile all the background information suggesting that the AG-induced antiglycation effect could also be significant in some plant physiological responses. Sugars are really important and crucial molecules not only in response to environmental stress conditions but also in growth and survival. Sugar signaling provides a way to optimally adjust the energy balance and the C:N ratio. It is also well-known that leaf senescence can be triggered by increasing sugar content. Reducing sugars can react spontaneously, albeit slowly, with proteins and lipids, leading to the formation of irreversibly cross-linked macroprotein derivatives known as advanced glycation end products (AGEs) [45]. These compounds threaten animal and plant health because they modify proteins. A systematic study of selective protein glycation, which is a non-enzymatic post-translational modification, was published previously [62], and the regulatory role of glycated plant proteins was related to sugar signaling [63]. The abundance of glycated proteins is variable under different stress conditions, such as light stress, heat stress, diurnal variation, and drought conditions. Kosová et al. [64] reviewed the proteoforms in plant proteomes under environmental stress conditions, providing a summary of protein glycation and also protein NO modifications, such as S-nitrosylation and Y-nitration.

The little-known glycation process is suggested as one of the non-enzymatic post-translational protein modifications which are supposed to contribute to the functional impairment of the plant proteome. Reducing sugars containing a free aldehyde or ketone group, such as glucose, fructose, and galactose, react with the N-terminal and lysine side chain amino groups of proteins. Reactive dicarbonyls are arginine residue-directed glycating agents, forming AGEs. A dominant dicarbonyl is a methylglyoxal formed through the Calvin cycle of photosynthesis. Concentrations of glucose, as well as methylglyoxal, one of the major AGEs, can be different and vary with age in plants; they could be dependent on the developmental stage (senescence), and certain biotic and abiotic stresses can also induce the change in their concentrations. Based on the proteomic analysis, it is suspected that there is an enrichment of the amino acid residue targets of glycation, arginine, and lysine residues, in predicted functional sites of the plant proteome, demonstrating the susceptibility of proteins to functional inactivation by glycation [65]. Aging is a glycation-related process in animals and plants [66]. Protein carbonylation and glycation play important roles in legume nodules [67]. Chaplin et al. profiled AGE products and revealed abiotic-stress-specific target proteins in *Arabidopsis* [68]. Moreover, protein glycation (especially monosaccharide autoxidation) was reported under conditions of osmotic stress in *Arabidopsis thaliana* [69]. Recently, Bilova and her colleagues identified important glycation hotspots in the *Arabidopsis* proteome and showed that these are age-dependent [70], although later in the same plant species, Put was the only PA identified as a candidate glycation hotspot in plant aging [71]. Thus, modification and fine-tuning of PA metabolism may lead to an efficient strategy for biotechnological approaches to boosting crop plant growth in the future. 

Further clarification of PA-induced glycation hotspots will add value to this research topic. Most recently, Dumont and Rivoal [72] observed the consequences of oxidative stress on glycolytic and respiratory metabolism and suggested the importance of oxidative stress-induced modifications in the central carbon metabolism of plants. Dobi et al. [73] similarly emphasized the role of AGEs in ROS production in human cells, and the mechanisms and targets of glycation and advanced glycation end products have been studied in simple eukaryotic models [74]. In another recent study, Amthor et al. developed engineering strategies that may boost crop productivity by limiting respiratory carbon loss [75].

Glycated proteins have conflicting yet important roles in the nutrition [76]. Food allergies are strongly associated with Maillard reactions following consumption of some pulses, such as chickpeas [77]. Some food processing steps can also influence Maillard reactions and, thereby, the immunogenicity and allergenicity of food proteins [78]. Hence, whereas some dietary plants and their nutritional content can contribute to aging processes [79], many edible and medicinal plants are important sources of efficient anti-AGE compounds [80]. 

It is widely studied that since AG possesses a nucleophilic hydrazine and a guanidine moiety, this compound may scavenge dicarbonyl compounds and prevent the formation of AGEs [45]. Accordingly, published experiments report the use of AG as a reference compound in tests of anti-AGE effects of plant extracts [81]. 

As regards its antiglycation effects, it is suggested in some studies that AG prevents the formation of AGEs by trapping dicarbonyl compounds [82]. Chelation is a common characteristic of such compounds as AG with multiple functional groups [83]. AG also can inhibit ascorbate oxidation at 1–5 mmol/L concentration and can positively affect the antioxidant defense responses, but elucidation of the details necessitates further research. Overall, it can be concluded that AG could be useful as an AGE inhibitor in plants, but more studies need to be conducted in order to decipher the exact mode of its action on glycation processes. 

## 5. Conclusions

In this review, we have collected some important evidence and discussed a number of examples showing that AG has different targets in plants that are related to PA catabolism. We critically discuss recent data showing that AG could act not only as a DAO inhibitor but also as a hypothetical NOS inhibitor and AGE chelator. These observations illustrate the importance of considering the multiple effects of AG to understand these controversial effects of AG in plants. The actual mechanisms underlying the AG effects require more precise methods and application systems to study those responses which are responsible for its effects on growth and development or differences in stress tolerance. Several studies have provided important insights into AG-related processes but lack information on the complex PA-NO interplay involving many signal pathways. Based on the examples mentioned above, in the future, AG should be regarded as a modulator of PA metabolism rather than a specific inhibitor of DAO in plants. These observations illustrate the crucial importance of understanding that AG is a multifunctional compound that can modify PA degradation by inhibiting DAO and, at the same time, it can fine-tune NO–PA interactions by inhibiting NOS-like activities. Moreover, the least known role of AG should be deciphered by experiments, as AG could inhibit protein glycation by trapping AGEs in plants, thus contributing to the elimination of methylglyoxal-induced oxidative stress; however, the protein glycation-related processes should be investigated with modern biotechnological approaches, for instance, RNASeq or other techniques (such as metabolomic analysis and chemical modification). In order to obtain more insights into the mode of action of AG in plants, more focused and well-structured chemical analyses are required in many plant species. Finally, it will be interesting to see whether the different targets discussed in this review are equally relevant in different cultivars, developmental stages, stress conditions, or application methods, enhancing our understanding of the effect of AG in plant abiotic stress responses. 

## Figures and Tables

**Table 1:** Summary of references related to DAO inhibitor activity of AG in plants.

Plant Species	Organ	AG Conc.	Treatment	Ref.
*Vicia faba*	seedling	5 mM	hypoxia-NaCl	[24]
*Hordeum vulgare*	leaf cell	10 µM	DILS ^1^	[19]
*Glycine max*	leaf	0.5–1 mM	salt stress	[25]
*Lycopersicum esculentum*	leaf disc	0–2 mM	salt stress	[22]
*Lupinus luteus*	seedling	1 mM	salt stress	[26]
*Nicotiana benthamiana*	leaf	0.5 mM	development	[20]
*Triticum aestivum*	seedling	0.5 mM	PAs-induced growth inhibition	[21]
*Cicer arietinum*	seedling	1 mM	wound healing	[27]
*Mesembryanthenum crystallinum*	root	1 mM	exogenous cadaverine	[28]
*Nicotiana tabacum*	leaf disc	2 mM	biotic stress	[23]
*Solanum lycopersicum*	seedling	1 mM	germination	[29]
*Medicago truncatula*	nodules	0.5 mM	nodulationsalt stress	[30]

^1^ DILS means dark-induced leaf senescence.

**Table 2:** References relating to NOS inhibitor activities of AG in plants.

Plant Species	Organ	AG Conc.	Stress Type	Ref.
*Capsicum annuum*	mature plant	2 mM	low temperature	[54]
*Arabidopsis thaliana*	root	2 mM	salt stress	[55]
*Helianthus annuus*	hypocotyl	5 mM	high temperature stress	[56]
*Nicotiana benthamiana*	crude extract	0–50 µM	TMV inoculation	[57]
*Zea mays*	seedling	3 mM	growth	[58]
*Olea europaea*	leaf	1 mM	salt/nitrosative stress	[59]
*Pisum sativum*	leaf extract	1 mM	abiotic stress	[60]

## Data Availability

Not applicable.

## Abbreviations

ABA	abscisic acid
ACC	aminocycloparopane carboxylic acid
ACC ox.	ACC oxidase
ACC synt.	ACC synthase
ACL5	T-Spm synthase
ADC	arginine decarboxylase
AG	L-aminoguanidine
AGEs	advanced glycation end products
Arg	L-arginine
Cad	cadaverine
CuAO	copper amine oxidase
dcSAM	decarboxylated SAM
DAO	diamine oxidase
DILS	dark-induced leaf senescence
GABA	gamma amino butyric acid
H_2_O_2_	hydrogen peroxide
iNOS	inducible NO synthase
NO	nitric oxide
NOS	nitric oxide synthase
ODC	ornithine decarboxylase
Orn	L-ornithine
PA	polyamine
PAO	polyamine oxidase
Pro	proline
Put	putrescine
ROS	reactive oxygen species
SAM	S-adenosylmethionine
SAMDC	SAM decarboxylase
Spd	spermidine
SPDS	Spd synthase
Spm	spermine
SPMS	Spm synthase
T-Spm	thermospermine
TCA cycle	tricarboxylic acid cycle
TPQ	2,4,5-trihydroxyphenylalanine quinone
